# Severe Macular Edema in Patients with Juvenile Idiopathic Arthritis-Related Uveitis

**DOI:** 10.1155/2013/803989

**Published:** 2013-08-19

**Authors:** Maria Pia Paroli, Claudia Fabiani, Giovanni Spinucci, Irene Abicca, Alfredo Sapia, Leopoldo Spadea

**Affiliations:** ^1^Department of Ophthalmology, Ocular Immunovirology Service, Sapienza University of Rome, Viale del Policlinico 155, 00161 Rome, Italy; ^2^Department of Biotechnological and Applied Clinical Sciences, Eye Clinic, University of L'Aquila, Italy

## Abstract

*Purpose*. To report the onset of severe macular edema in adolescent female patients affected by juvenile idiopathic arthritis (JIA). *Methods*. Four female patients affected by JIA-related chronic anterior uveitis (CAU), complicated by severe macular edema, were retrospectively analyzed. Macular area was evaluated by fluorescein angiography and optical coherence tomography (OCT). *Results*. CAU was bilateral in three patients. Mean age of uveitis and arthritis onset was, respectively, 4.5 ± 1.7 years and 6.0 ± 3.9 years. All patients underwent cataract extraction surgery. Despite ocular inflammation being controlled by topical/systemic therapy, during adolescence (mean age of appearance/diagnosis: 12.7 ± 3.9 years) patients developed severe unilateral macular edema. OCT revealed massive macular thickening (range from 550 **μ**m to 1214 **μ**m). *Conclusions*. Macular edema appeared in female adolescent patients in eyes with long-dating CAU submitted to cataract surgery. In such patients, in presence of age-related microvascular changes due to the enhancer effect of sex hormones, cataract extraction should be a factor triggering the retinal complication.

## 1. Introduction

Eye involvement in juvenile idiopathic arthritis (JIA) is frequent: typically the uveitis is a chronic, bilateral, and nongranulomatous anterior uveitis with insidious and asymptomatic course. Macular edema is one of the most sight-threatening complications, ranging from 3% to 47% [1]. It is reported that macular edema is the cause of legal blindness in 8% of children affected by active uveitis [1]. Pathophysiology of inflammatory macular edema is still unclear. The major cause might be the breakdown of the inner blood-retinal barrier. Microvascular factors are involved and probably enhanced by inflammatory factors and by sex hormones. In our study, we describe four peculiar cases of severe macular edema which appeared during adolescence in JIA female patients affected by chronic anterior uveitis (CAU) from infancy, in which uveitis was under control for a long time. 

## 2. Material and Methods

The clinical history of four patients affected by JIA-related uveitis presenting highly severe macular  edema was retrospectively analyzed. Fluorescein angiography (FA) and optical coherence  tomography (OCT) examination were also performed to determine qualitatively and quantitatively  the entity of macular involvement. This study was conducted according to the principles of the  Helsinki Declaration and good clinical practices. The following data were collected  for each patient: age at first visit, age at onset of uveitis, age at onset of arthritis, clinical features of  uveitis, and medical and surgical treatment. Each patient had complete eye examinations including  visual acuity examination by Snellen charts or illiterate E charts, slit-lamp biomicroscopy, intraocular pressure, and fundus evaluation. Moreover, each patient underwent a routine laboratory  examination including complete blood cell count, erythrocyte sedimentation rate, urinalysis and  serum biochemical analysis (e.g., autoantibodies, HLA I and II class typing). Fluorescein  angiography using the digital imaging system Topcon IMAGEnet H 1024 (Topcon, Inc., Paramus, NJ, USA) was performed in only one patient (Case 1), while the other ones refused  to do the examination. Macular morphology and thickness were studied by Spectralis OCT (Heidelberg Engineering, Heidelberg, Germany) which provides up to 40.000 A scans/sec with a  depth resolution of 7 *μ*m and a transversal resolution of 14 *μ*m. The retinal thickness measurements were obtained through a 20 × 15 raster scans, consisting of 19 high-resolution line scans. Retinal  thickness values were calculated for nine areas, corresponding to the Early Treatment Diabetic  Retinopathy Study (ETDRS) areas. Standard radial scans of macular region were also performed to  assess macular morphology and thickness.

## 3. Results

Four female adolescent patients affected by CAU presented severe macular edema after a mean of  12.7 ± 3.9 years from the diagnosis. Chronic anterior uveitis was bilateral in three patients and unilateral in one. Mean age of uveitis onset was 4.5 ± 1.7 years (range from 2 to 6); mean age of  arthritis onset was 6.0 ± 3.9 years (range from 3 to 7). All patients underwent cataract extraction  surgeries. Two patients underwent bilaterally pars plana lensectomy with anterior vitrectomy (Cases 2 and 3). Two patients underwent unilateral cataract surgery by phacoemulsification (Cases 1 and 4): only in one of these an intraocular lens (IOL) was implanted (Case 4). Despite ocular  inflammation being well controlled by topical and systemic steroids and/or immunosuppressors,  some years later, during adolescence (11, 13, 15, and 16 years old, resp.), patients developed  unilateral sight-threatening severe macular edema. Mean visual acuity was 20/80, and OCT revealed  massive macular thickening with mean foveal thickness of 866 ± 537 *μ*m (range 550 to 1214).   Mean follow-up time was 8.5 ± 1.1 years. The clinical history and the clinical progress of ocular  inflammation of these 4 patients affected by JIA-related CAU, complicated by severe cystoid macular edema (CME), were analyzed.

**Case 1**

An 8-year-old girl was referred to us. Both pauci-articular JIA and unilateral uveitis were diagnosed at the age of two year. The young patient previously treated with oral and topical  steroids presents a corrected distance visual acuity (CDVA) of 20/20 in both eyes.   Biomicroscopy revealed band keratopathy, keratic precipitates, posterior synechiae, and clear lens in right eye. Fundus examination disclosed no pathological alterations in both eyes.   During our followup, the patient was treated with topical desametazone, oral prednisone (0.5 mg/Kg/day followed by gradual tapering of the dosage) and hydroxychloroquine (400 mg/day). Visual acuity was gradually impaired in the right eye because of complicated cataract, which was removed at the age of 12 by phacoemulsification without IOL implantation.   A complete visual recovery was then obtained.   During the following months anterior uveitis relapsed, and episodes responded well to topical steroid treatment. At the age of 16, CDVA in the right eye impaired at 20/200 and CME was diagnosed and confirmed by FA and OCT examination (foveal thickness 700 *μ*m). Oral prednisone was added (0.5 mg/Kg/day). Due to poor response to systemic steroid treatment, periocular steroid injections was advised, but the patient refused. At the end of follow-up CDVA was 20/250 in the right eye. CDVA of 20/20 in the left eye was preserved and OCT showed normal retinal thickness.

**Case 2**

**Figure 1:**
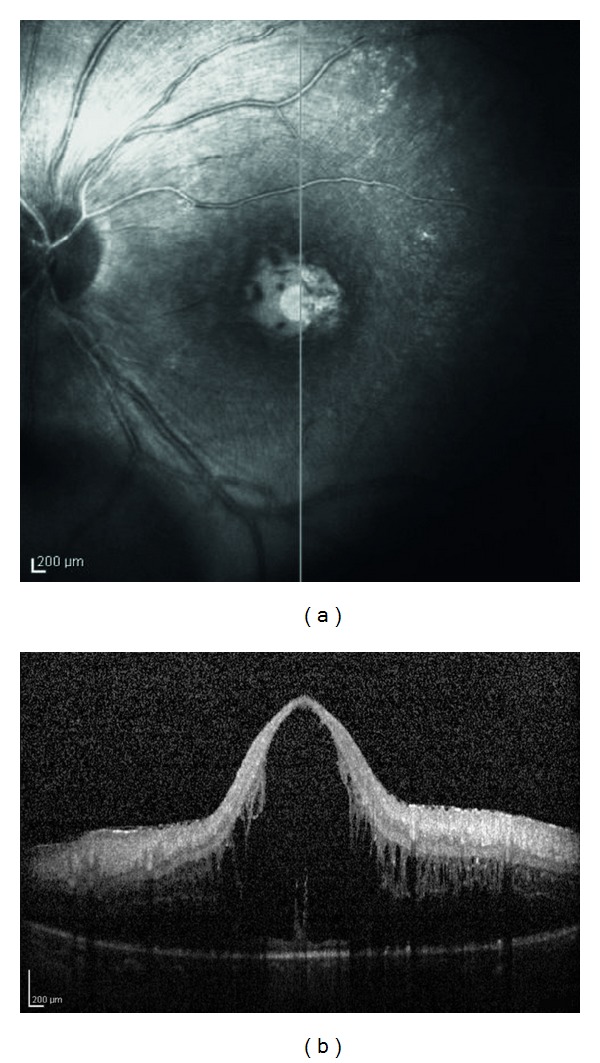
Patient no. 2: left eye. Scanning laser ophthalmoscope fundus image (on the left) showing the position of optical coherence tomography (OCT) section. Spectralis OCT (on the right) shows severe macular edema (foveal thickness 1160 *μ*m).

A 7-year-old girl was referred to us for bilateral CAU since the age of 6; 2 years later, articular involvement occurred, and a diagnosis of pauciarticular JIA was performed. CDVA at presentation was 20/20 in right eye and 20/20 in left eye. Ocular examination disclosed bilateral band keratopathy, seclusio pupillae, posterior subcapsular cataract, and no retinal alterations. During the followup, the patient presented with relapses of anterior uveitis in the left eye, while the right eye showed milder uveitis activity. Treated with topical desametazone and oral prednisone (0.5 mg/Kg/day followed by gradual tapering of the dosage), visual acuity was gradually impaired bilaterally due to cataract evolution. Patient underwent cataract extraction by pars plana lensectomy with anterior vitrectomy in left eye at the age of 8 and 9 months later in right eye, achieving complete bilateral visual recovery. One year later, CDVA reduced in the left eye because of relapsing uveitis. At the age of 11, CME was diagnosed in left eye and confirmed by OCT (Figure 1) and FA, poorly responding to repeated peribulbar injections of metilprednisolone and oral prednisone (1 mg/Kg/day followed by gradual tapering of the dosage). CDVA at followup was 20/40 in the left eye and 20/20 in the right one; the OCT showed mild retinal thickness.

**Case 3**

**Figure 2:**
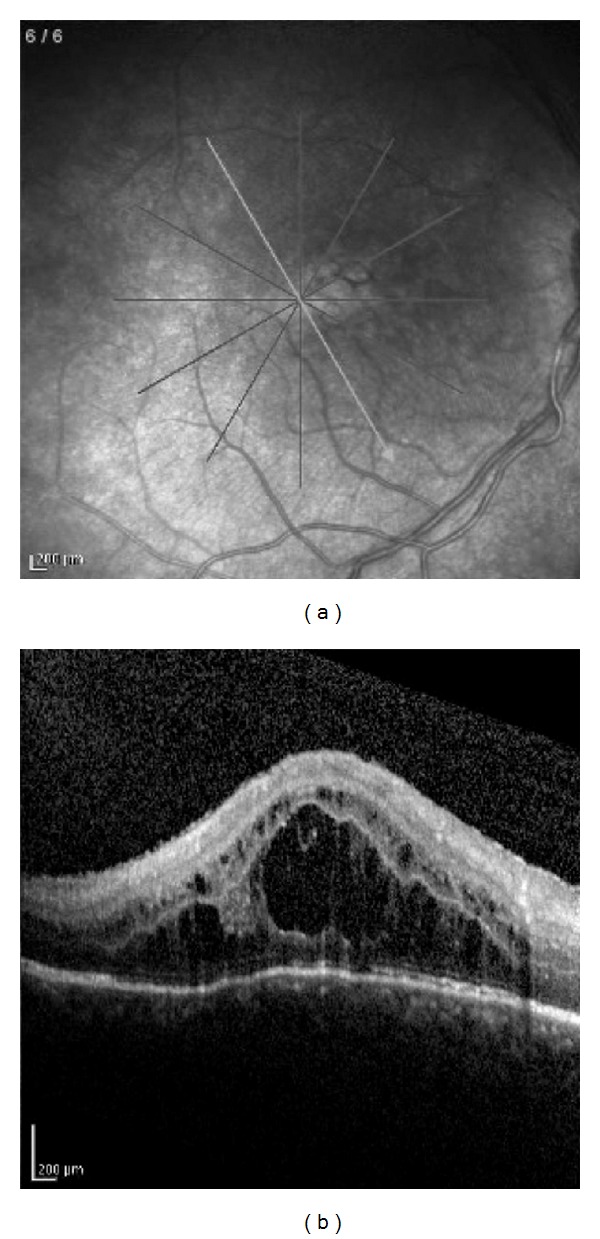
Patient no. 3: right eye. Scanning laser ophthalmoscope fundus image (on the left) showing the position of optical coherence tomography (OCT) section. Spectralis OCT (on the right) shows severe macular edema (foveal thickness 947 *μ*m).

A 5-year-old girl was referred to us with recently diagnosed asymptomatic bilateral CAU. At presentation, CDVA was 20/600 in both eyes. Ocular examination disclosed bilaterally band keratopathy, seclusio pupillae, corticonuclear and posterior subcapsular cataract, vitreous haze, vitritis, no retinal changes. During the followup, treated with topical desametazone and oral prednisone (0.5 mg/Kg/day followed by gradual tapering of the dosage), CDVA was gradually impaired bilaterally due to cataract evolution. Articular involvement occurred when patient was 11 years old: diagnosis of pauci-articular arthritis was performed. Patient underwent pars plana lensectomy, with anterior vitrectomy, at the age of 7 years, in the right eye and one year later in the left eye with complete bilateral visual recovery. At the age of 12 arthritis relapsed and patient treated with cyclosporine (200 mg/day), methotrexate (15 mg/day) and oral prednisone (0.2 mg/Kg/day followed by gradual tapering of the dosage); starting from the age of 13, she was treated with intravenous infusions of anti-TNF biological agents (Infliximab). At the age of 12, CME was diagnosed in right eye and confirmed by OCT (Figure 2), poorly responding to repeated peribulbar injections of metilprednisolone and oral prednisone (1 mg/Kg/day followed by gradual tapering of the dosage). CDVA at the end of followup was 20/200 in right eye and 20/20 in left eye, and OCT showed normal retinal thickness.

**Case 4**

**Figure 3:**
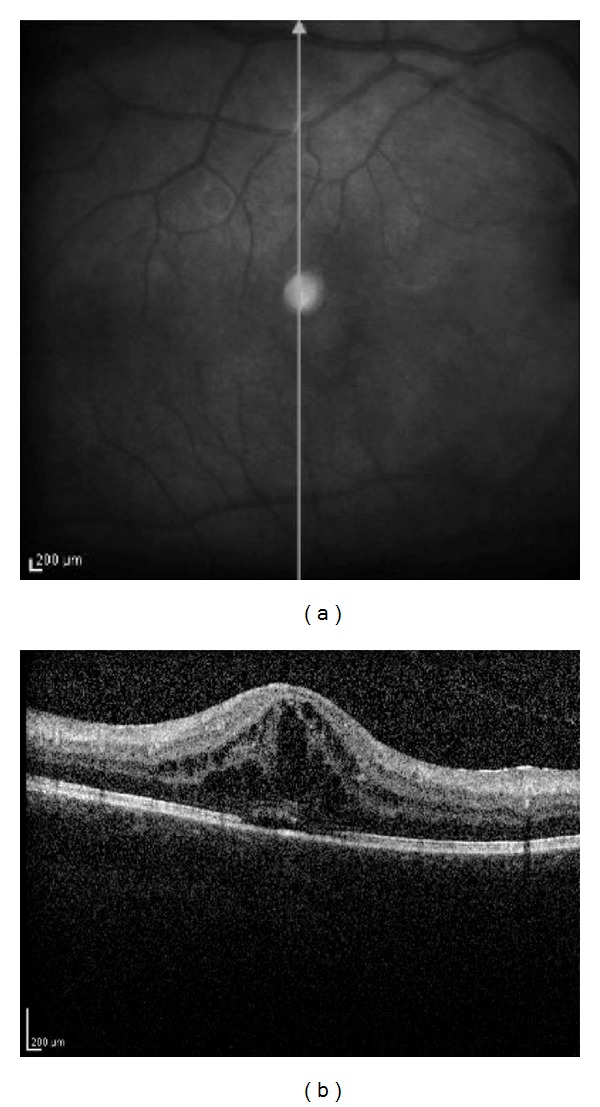
Patient no. 4: right eye. Scanning laser ophthalmoscope fundus image (on the left) showing the position of optical coherence tomography (OCT) section. Spectralis OCT (on the right) shows severe macular edema and a foveal detachment (foveal thickness 600 *μ*m).

A 5-year-old girl was referred to us for recently diagnosed bilateral CAU associated with pauciarticular JIA with onset one year before. At presentation, CDVA was 6/20 in right eye and 20/20 in left eye. Ocular examination disclosed band keratopathy, keratoprecipitates, anterior chamber flare, seclusio pupillae, transparent lens, normal optic disk and retina in right eye, and few cells (1+) in anterior chamber in left eye. No pathological alterations were described in both eyes. During the followup, patient was treated with topical desametazone and oral prednisone (0.5 mg/Kg/day followed by gradual tapering of the dosage). Visual acuity was gradually reduced in right eye because of cataract evolution; therefore, the patient underwent phacoemulsification with IOL implantation at the age of 10 years; few days after surgery was uveitis relapsed in right eye with anterior chamber flare and vitritis. Patient has been treated with topical desametazone, peribulbar metilprednisolone, and systemic prednisone; visual acuity was restored in two months (20/20). At the age of 12, CME was diagnosed in right eye and confirmed by OCT examination (Figure 3) and FA, poorly responding to repeated peribulbar injections of metilprednisolone and oral prednisone (1 mg/Kg/day followed by gradual tapering of the dosage). Intravitreal injections of bevacizumab have been proposed, but her family refused this treatment. At the end of followup, CDVA was 6/20 in right eye and 20/20 in left eye. Normal retinal thickness was detected by OCT in the left eye.

## 4. Discussion

The peculiarity of these four reported cases is that cystoid macular edema appeared in adolescent female patients in eyes with long-dating uveitis, developing after a mean time of 3.25 ± 0.90 years from cataract extraction. Pathogenesis of CME in inflammatory disorders is still unclear: malfunction of blood-retinal barrier plays a central role and several inflammatory and vasoactive peptides are probably involved. Moreover elevated levels of pro-inflammatory cytokines and vascular endothelial growth factors (VEGF) have been found [2]. 

Cataract extraction in eyes affected by uveitis may represent one of the most important factors in triggering CME; in fact, Kotaniemi and Penttilä reported macular edema in 44% of JIA patients after cataract surgery [3]. Our patients underwent cataract surgery when ocular inflammation was well controlled, and macular edema was observed several months after surgery. Macular edema usually occurs 4 to 12 weeks after cataract surgery; sometimes it is a very late complication and appears some years after surgery. Cataract extraction stimulates ocular inflammation by releasing pro-inflammatory mediators, for example, prostaglandins, which diffuse throughout posterior chamber and provoke macular edema. However, this complication may be also interpreted as the result of vitreous traction during surgery. Concerning our patients, bilateral complicated cataract was removed by pars plana lensectomy and anterior vitrectomy without IOL implantation in Cases 2 and 3: CME developed only in one eye. In Cases 1 and 4, unilateral complicated cataract was removed by phacoemulsification, with (Case 4) or without (Case 1) IOL implantation; CME developed only in the eye treated by surgery. 

In all eyes, it developed after a mean time of 3.25 years from cataract extraction: in 2 patients, macular involvement developed 24 to 36 months after surgery; in the remaining 2 patients it developed after 48 to 60 months. Although in our patients only the eyes submitted to surgery were the interest of by CME, cataract extraction does not have to be considered the only triggering factor leading to macular edema. In fact, this mechanism seems to be insufficient to explain alone unilateral edema in the two cases submitted to bilateral surgery. In contrast to postoperative CME due to ocular surgery, where the noxious influence is of limited duration, the release of inflammatory mediators in course of uveitis is more chronic and will invariably perpetuate the retinal damage. In patients with complicated JIA-related CAU, remission rarely occurs and needs chronic therapy [4]. 

In the reported four young patients, CME developed in eyes in which CAU lasted for more than 6 years: inflammatory macular edema can be actually related to uveitis duration because of long-acting inflammatory stress on macular cells. Severity of CAU should have played a role in Case 2 in which CME developed in the eye where relapses were more frequent [5]. Macular edema is a well-recognized complication of JIA-related CAU [6].

Furthermore, in each case CME appears to have been preceded by a relapse of the uveitis, and active uveitis is a well-recognized risk factor for macular edema.

Appearance of maculopathy during adolescence might be due to the enhancer effect of sex hormones inducing microvascular changes, as previously described for diabetic retinopathy. In fact, Chaurasia et al. disclosed a higher serum level of follicle-stimulating hormone in diabetic patients with retinopathy than in those without retinopathy or in healthy subjects [7]. VEGF is closely associated with diabetic retinopathy: upregulation causes loosening of tight junctions, vascular leakage, and macular edema. Sex hormone changes have been demonstrated to promote the worsening of diabetic retinopathy by upregulating VEGF levels; in fact, experimental lines of evidence suggest that estrogens increase the VEGF expression.

Moreover, estrogens have been supposed to promote choroidal neovascularization by increasing VEGF receptors [8].

Molecular mechanisms by which sex hormones modulate inflammation are not still fully understood; proinflammatory effect has been proposed as well as anti-inflammatory activity. The hypothalamus-hypophysis-gonadal axis may be involved in autoimmune disorders, and the influence of hormones on inflammatory cytokines in autoimmune disorders and particularly in uveitis needs further investigation. Several studies provided evidence of the modulatory effect of the estrogens on the CD4 cells subpopulation designed Th1 and Th2. Sanghvi et al. demonstrated that anterior acute uveitis is partially dependent on the levels of either estrogen or progesterone or both [9]. The withdrawal of the proven anti-inflammatory effects of these hormones may provoke the onset of uveitis itself. Studies on behaviour of noninfective uveitis during pregnancy showed uveitis recovery during mid and late pregnancies and flare up in postpartum period, like in other autoimmune diseases. Further studies about the interactions of neuroendocrine, immunologic, and microvascular factors involved in inflammatory macular edema are necessary, together with deepened investigations on the hormonal status of patients with JIA and CAU to prevent cystoid macular edema in young affected patients.

## 5. Conclusions

Severe macular edema appeared in female adolescent patients in eyes with long-dating chronic anterior uveitis submitted to cataract surgery. In such patients, in presence of age-related microvascular changes due to the enhancer effect of sex hormones, cataract extraction should be a factor triggering the retinal complication.
